# Occurrence and characterisation of *Salmonella enterica* subspecies *diarizonae* serovar 61: k: 1, 5, (7) in sheep in the federal state of Thuringia, Germany

**DOI:** 10.1186/s12917-018-1741-4

**Published:** 2018-12-17

**Authors:** Ulrich Methner, Udo Moog

**Affiliations:** 1 Institute of Bacterial Infections and Zoonoses at the Friedrich-Loeffler-Institute, Federal Research Institute for Animal Health, Naumburger Str. 96a, 07743 Jena, Germany; 2 Thuringian Animal Diseases Fund, Victor-Goerttler-Str. 4, 07745 Jena, Germany

**Keywords:** *Salmonella diarizonae*, Sheep, Germany, Epidemiology, Zoonotic potential

## Abstract

**Background:**

The occurrence of *Salmonella enterica* subspecies *diarizonae* serovar 61: k: 1, 5, (7) (SA*S*d) and other *Salmonella* organisms in sheep in the German federal state of Thuringia was examined for the first time. Pooled faecal samples from 90 flocks located in this state were monitored.

**Results:**

Only SA*S*d was detected in 74 (82.2%) out of the 90 sheep herds, other *Salmonella* serovars were not identified. A positive correlation was found between the flock size and the detection probability of SA*S*d. Despite the agent’s high prevalence, clinical symptoms of a disease exclusively due to SA*S*d have not been observed. The SA*S*d strains were characterised by macrorestriction analysis, antimicrobial testing and the biochemical profile. All strains were sensitive to 13 out of 14 antimicrobial substances and resistant to only sulfamethoxazole. The high number of macrorestriction groups of SA*S*d strains indicated a low clonality of the serovar.

**Conclusions:**

Data from sheep derived foods and public health data in Germany strongly suggest that the significance of SA*S*d for public health is considerably lower than that of serovars belonging to *Salmonella enterica* subspecies *enteri*ca. For this reason and because of the low disease-causing potential of SA*S*d in sheep, it is worthwile to consider a reduction in ongoing activities from combating to monitoring serovar 61: k: 1, 5, (7) in the sheep population.

## Background

Sheep can be infected with a wide range of *Salmonella enterica* serovars that are not restricted to ovine and do not establish an endemic pattern [1]. The host-specific serovar *Salmonella enterica* subspecies *enterica* serovar Abortus-ovis represents a common cause of abortion and mortality in young lambs in endemic areas of Europe and Western Asia [1, 2]. While the sheep associated serovar *Salmonella enterica* subspecies *diarizonae* serovar 61: k: 1, 5, (7) (SA*S*d) is also considered as host-adapted [3–6], it displays a very different epidemiological pattern. SA*S*d is able to produce both intestinal and extra-intestinal infections with faecal, vaginal and nasal colonisation as well as shedding, but mostly without clinical disease [7–9]. However, it has also been reported that SA*S*d may occasionally cause rhinitis or chronic nasal inflammation [10, 11], orchitis [12], or aborted foetuses [4]. As these properties deviate from the classical characteristics of host-restricted, host-adapted or ubiquitous serovars [13], the term sheep associated serovar [6] appears most appropriate to characterise serovar 61: k: 1, 5, (7).

A few studies have been undertaken to investigate the prevalence of SA*S*d and other *Salmonella* serovars in sheep, particularly in the United Kingdom [5, 14], Sweden [6], Norway [9], USA [15], Iceland [16] and Switzerland [17]. Among these countries, SA*S*d prevalence rates in sheep flocks varied from 11% [17] to more than 70% [15] which is very likely due to different study designs. Nevertheless, SA*S*d was the predominant serovar in all these countries [6, 9, 14, 15, 17] except for Iceland [16]. Data specifying the occurrence of *Salmonella* spp. or SA*S*d in the German sheep population are not available.

Therefore, the aim of this study was to obtain information on the occurrence of *Salmonella* spp. in sheep throughout the federal state of Thuringia. Furthermore, the isolated *Salmonella* organisms were characterised phenotypically and genotypically to explore a possible epidemiological connection.

## Methods

### Sampling of sheep flocks and bacteriology

**Fig. 1:**
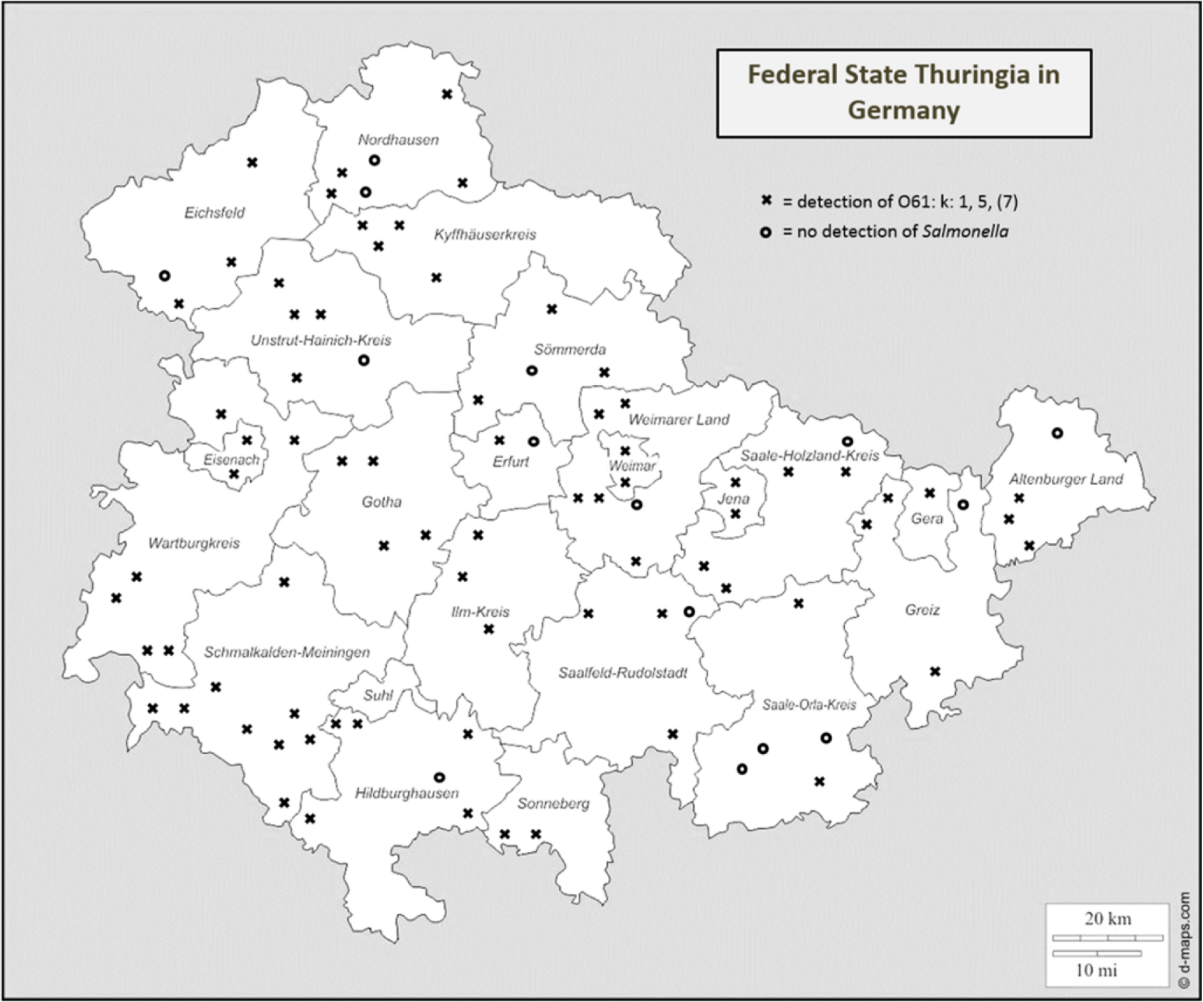
Location of sheep flocks sampled for *Salmonella* in the federal state Thuringia in Germany. (source: https://d-maps.com/carte.php?num_car=24110&lang=de)

A total of 90 sheep flocks of different size (9 categories of herd size) located throughout the German federal state of Thuringia (Table 1, Fig. 1) were monitored over a period of 1 year. Herds from each Thuringian region were selected, which are representative in terms of sheep density, size and management system. In each herd, 3 pooled faecal samples (ca. 25–30 g) consisting each of fresh sheep faeces from at least 10 different places, in order to represent at least 10 different animals and several parts of the herd were collected (in total 270 samples from 90 herds). Individual samples were either taken rectally or collected from fresh droppings without environmental contamination. The pooled samples were kept in plastic containers until bacteriological examination started on the day of sampling. Analysis of faecal samples was carried out according to ISO 6579-1 [18]. Buffered peptone water, modified semi-solid Rappaport-Vassiliadis, xylose lysine deoxycholate agar and Rambach agar were applied for the analysis. Furthermore, deoxycholate-citrate agar was used to possibly increase the isolation rate of SA*S*d (all from SIFIN, Berlin Germany). All *Salmonella* isolates were serotyped using poly- and monovalent anti-O as well as anti-H sera (SIFIN) according to the Kauffmann-White scheme [19]. The biochemical profile of *Salmonella* strains originating from different regions was determined using the identification system API 20E (bioMerieux, Nürtingen, Germany). Furthermore, sheep flocks were also observed for signs of morbidity and the history of clinical symptoms in the flock was assessed.

**Table 1:** Occurrence of *Salmonella* serovar 61: k: 1, 5, (7) in sheep flocks of different size in the federal state Thuringia in Germany

Herd size (number of animals)	Number of herds sampled	Number of positive herds	% of positive herds
1–30	15	3	20.0
31–50	5	4	80.0
51–100	7	6	85.7
101–300	4	4	100
301–500	23	21	91.3
501–750	18	18	100
751–1.000	9	9	100
1.001–1.500	5	5	100
1.501–3.000	4	4	100
Total	90	74	82.2

### Antimicrobial susceptibility testing

Antimicrobial susceptibilities of the *Salmonella* strains detected in sheep flocks were assessed by determining the minimum inhibitory concentration (MIC) using the broth microdilution method with Sensititre™ EUVSEC plates (Trek Diagnostic Systems Ltd., East Grinstead, United Kingdom). Epidemiological cut-off values according to the European Committee on Antimicrobial Susceptibility Testing (EUCAST) were used [20]. Antimicrobial susceptibilities to sulfamethoxazole, trimethoprim, ciprofloxacin, tetracycline, meropenem, azithromycin, nalidixic acid, cefotaxime, chloramphenicol, tigecycline, ceftazidime, colistin, ampicillin and gentamicin were examined.

### Genotyping using pulsed-field gel electrophoresis (PFGE)

Macrorestriction analysis was carried out as described [21]. Isolates were considered to be different when a one-band difference between fragments over 70 kb was observed [21, 22].

### Statistical analysis

A logistic regression was performed between the two variables infection status as the dichotom dependent variable and herd size (category as nominal variable 1 to 9, Table 1) as independent variable. Statistical evaluation was conducted using MedCalc Software.

## Results

### Bacteriological analysis

Pooled faecal samples (*n* = 270) from 90 farms were examined for the occurrence of *Salmonella* organisms (Fig. 1). Only SA*S*d was detected in 74 (82.2%) out of the 90 sheep flocks, other *Salmonella* serovars were not found (Table 1). In total, 206 (76.3%) pooled samples were positive for SA*S*d and 64 (23.7%) were negative. The odds ratio for herd size was 2.285 with a 95% confidence interval from 1.878 to 2.779 (*p* < 0.0001). Therefore, the parameter herd size is a highly significant variable for the prediction of the infection status of the flock.

In nearly all flocks with more than 100 animals the sheep associated SA*S*d was detected. Indeed, even in flocks with only 30–100 animals, 80 -86% of the flocks were positive for SA*S*d. All herds with more than 500 animals showed a prevalence of 100%. The prevalence value of 100% for the herd size 101–300 is probably an outlier because only 4 herds were sampled. In all flocks with proven SA*S*d in faecal samples no signs of morbidity exclusively due to this serovar were observed and the history of the flocks did not reveal any evidence for clinical symptoms caused by SA*S*d.

### Antimicrobial susceptibilities

All 74 SA*S*d strains tested were sensitive to 13 out of 14 antimicrobials. The organisms were all resistant to sulfamethoxazole (MIC > 512 μg/ml). Level of susceptibility against the other antimicrobial substances did not reveal differences between the strains, the resistance patterns of the SA*S*d were nearly identical. MIC values (μg/ml) of the strains were as follows: trimethoprim (1–2), ciprofloxacin (0.06), tetracycline (4–8), meropenem (< 0.03), azithromycin (8–16), nalidixic acid (8), cefotaxime (< 0.25), chloramphenicol (< 8), tigecycline (1–2), ceftazidime (< 0.05), colistin (< 1), ampicillin (2) and gentamicin (1). Further differentiation among the SA*S*d strains based on the resistance pattern was not possible.

### Biochemical characterisation

SA*S*d strains were tested using API 20E and identified as *Salmonella.* They all showed a positive ONPG reaction due to their β-galactosidase production which is characteristic for *Salmonella enterica* subspecies *arizonae* and *diarizonae* [19] and might, however, complicate the isolation of SA*S*d organisms [22]. Also, tests for lysine decarboxylase, production of hydrogen sulphide and production of acid from sorbitol varied between the SA*S*d strains examined. Therefore, “Analytical Profile Indices” of the SA*S*d strains were I for 7,704,552 (lysine decarboxylase +), II for 3,704,552 (lysine decarboxylase -) and III for 3,704,152 (lysine decarboxylase -, sorbitol -) (Table 2). The different API 20E indices I, II, or III were not used to generate a further distinction in addition to the macrorestriction groups, since a correlation between both features was missing. Because of the biochemical characteristics of SA*S*d, the use of more selective media than required by ISO 6579-1 [19] (e.g. deoxycholate-citrate agar) very likely raised the number of SA*S*d strains found in sheep flocks.

**Table 2:** Characteristics of strains of *Salmonella* serovar 61: k: 1, 5, (7)

Strain	Analytical profile index	Macrorestriction pattern		Macro-restriction group^c^
		*Xba* I X^a^	*Spe* I S^b^	
1478	II	5	2	E
1513	II	1	4	A
1515	I	2	1	B
1520	I	2	1	B
1521	III	1	4	A
1529	II	3	2	C
1530	II	3	2	C
1544	II	5	2	E
1546	III	3	2	C
1547	III	3	2	C
1552	III	5	6	K
1561	II	6	2	L
1563	I	2	1	B
1569	II	4	5	D
1570	III	5	2	E
1575	II	9	2	M
1588	III	3	2	C
1641	III	5	2	E
1645	I	8	3	H
1646	III	3	2	C
1649	II	6	6	F
1650	III	7	2	G
1651	III	7	2	G
1674	II	8	3	H
1675	III	5	2	E
1676	II	9	3	I
1677	I	2	1	B
1679	I	2	4	J
1680	III	1	4	A

Biochemical index: I: 7704552 (lysine decarboxylase +), II: 3704552 (lysine decarboxylase -), III: 3704152 (lysine decarboxylase -, sorbitol -)

**Fig. 2:**
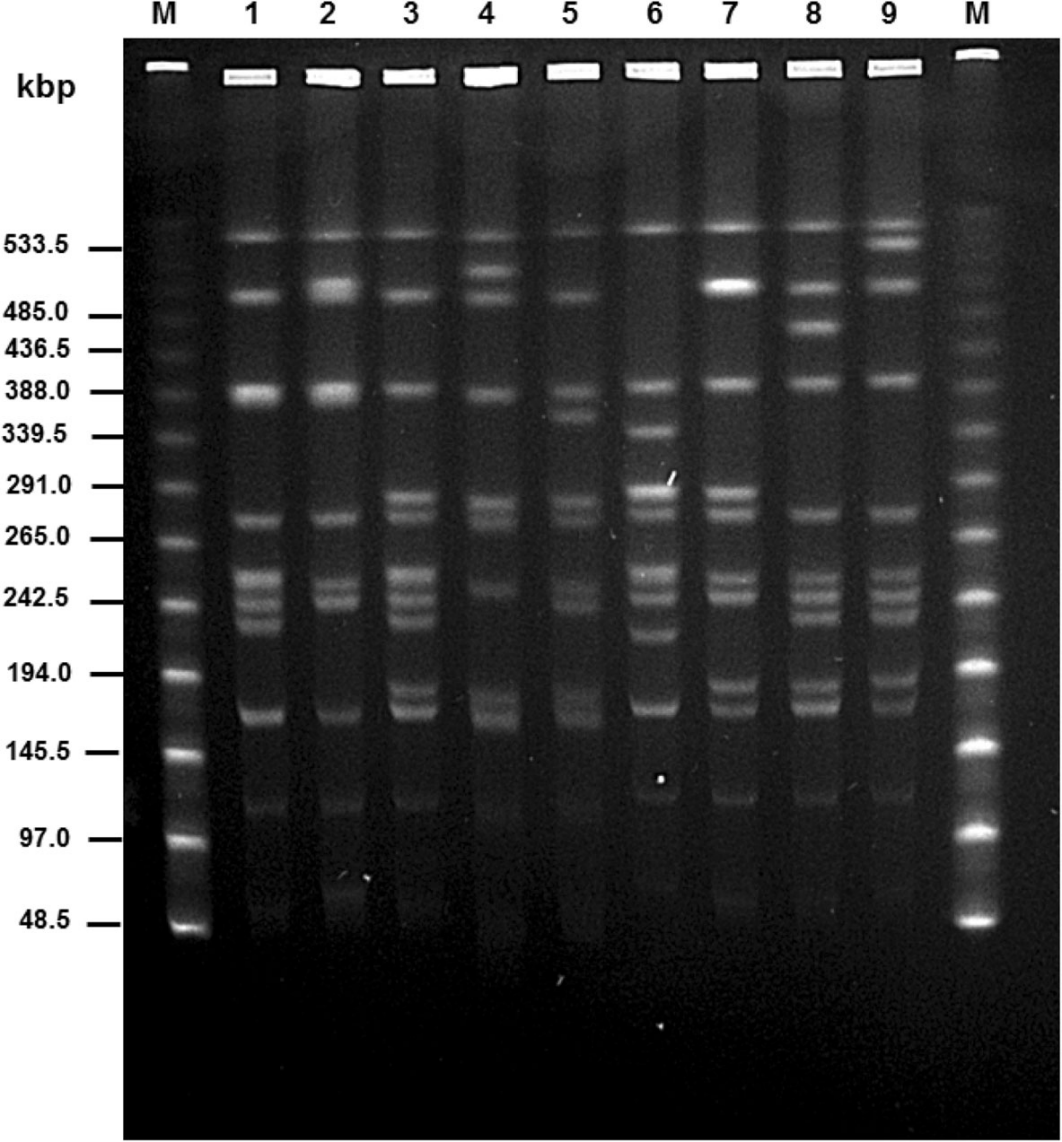
*Xba*I (X1-X9) macrorestriction patterns of *Salmonella* strains 61: k: 1, 5, (7)

^a,^Pattern numbers correspond to lane numbers in Fig. 2

**Fig. 3:**
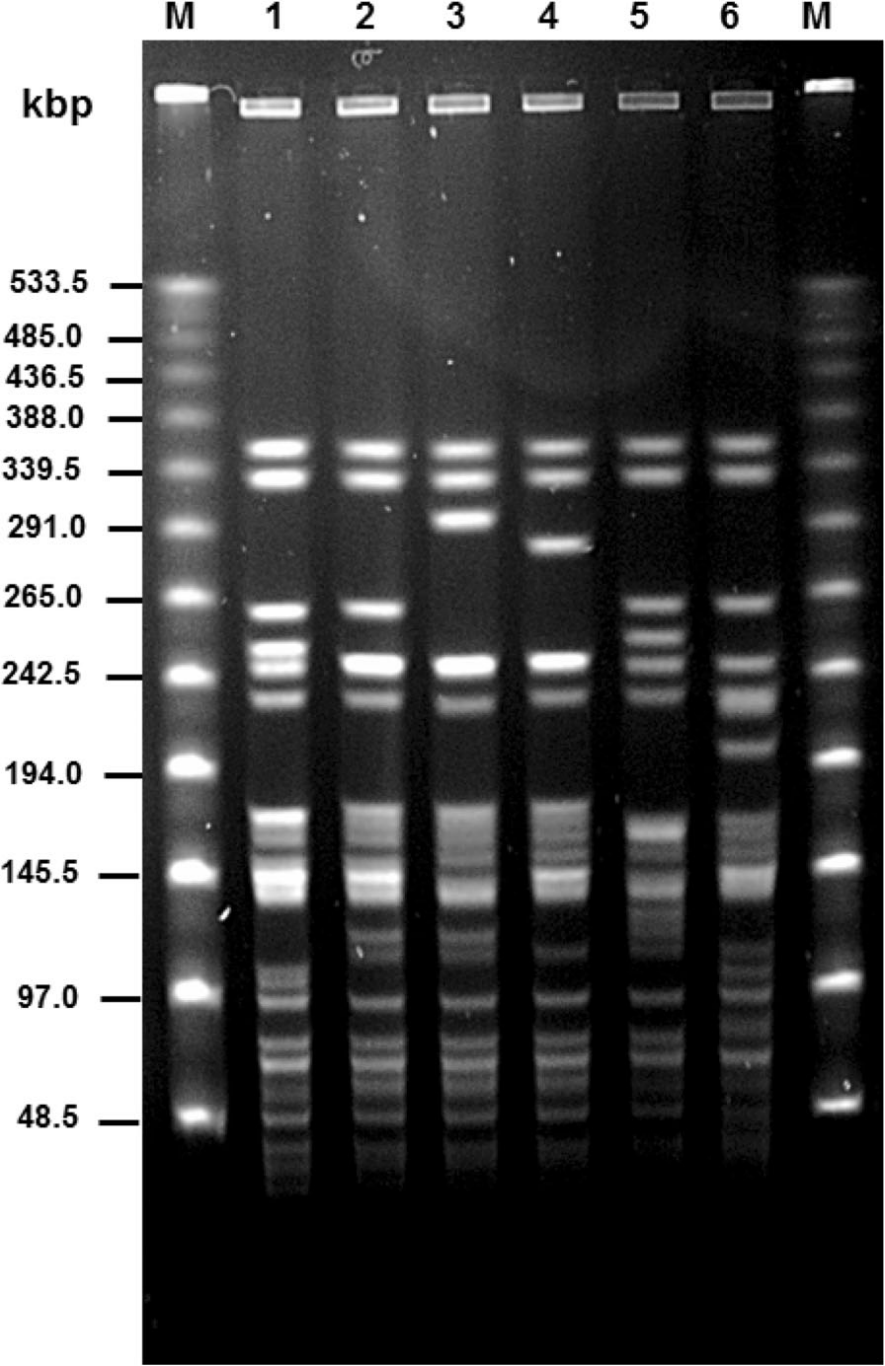
*Spe* I (S1-S6) macrorestriction patterns of *Salmonella* strains 61: k: 1, 5, (7)

^b^Pattern numbers correspond to lane numbers in Fig. 3

^c^Macrorestriction group as combination of macrorestricion patterns

### Macrorestriction analysis

**Fig. 4:**
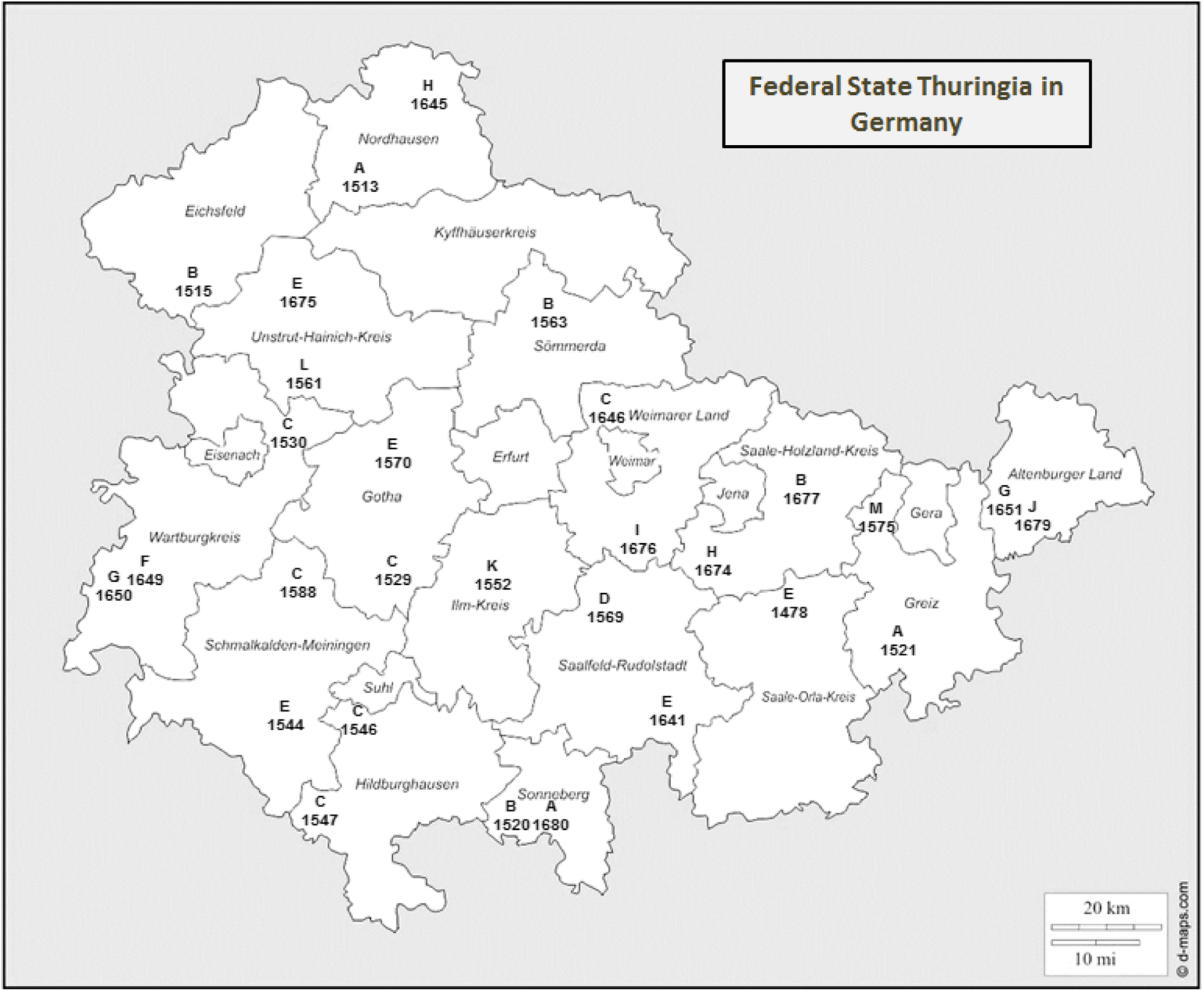
Distribution of macrorestriction groups (A-M) of *Salmonella* serovar 61: k: 1, 5, (7) strains in sheep flocks in the federal state Thuringia in Germany. (source: https://d-maps.com/carte.php?num_car=24110&lang=de)

Two different restriction endonucleases, *Xba*I and *Spe*I (Figs. 2 and 3) were used to cleave whole-cell DNA of 29 SA*S*d isolates originating from different regions and flocks in Thuringia (Table 2, Fig. 4). *Xba*I digestion yielded nine (X1 to X9) different patterns, *Spe*I digestion resulted in six (S1 to S6) patterns. Therefore, the SA*S*d strains tested revealed a high genetic diversity. Results of macrorestriction analysis allowed the assignment of the 29 strains examined in this study to 13 macrorestriction groups A to M (Table 2). In most cases, strains belonging to the same macrorestriction group (e.g. A for strains 1513, 1680 or B for strains 1515, 1520, 1563, 1677) were located apart from each other in very different regions of Thuringia. Only group C occurred more frequently in different related counties of the federal state (Fig. 4).

## Discussion

In the sheep population of the federal state of Thuringia, only the serovar 61: k: 1, 5, (7) was detected in more than 80% of the examined flocks. The dominant appearance of SA*S*d in sheep confirms study results from other countries. However, the detected prevalence of 82% in this study is high compared to data from other countries, such as Norway (20%) [9], Switzerland (11%) [17], USA (72%) [15] or Sweden (12–40%) [6]. Real differences probably exist between countries, but the variation will also be heavily influenced by different study designs, sampling methods or sampling material. In view of the moderate to high prevalence of SA*S*d in different countries, the occurrence of this serovar seems to be due to a long lasting persistence in sheep flocks because of its host associated characteristics. Other *Salmonella* serovars that are not confined to sheep are obviously rare and do not establish an epidemic pattern, and their occurrence appears to be related to pastures contaminated with wild bird faeces [2].

Despite the considerable detection rate of SA*S*d in sheep flocks, it might be possible that the real incidence in sheep is even higher because of the biochemical features of this serovar [14]. The lactose positive SA*S*d organisms might constitute a special diagnostic problem, therefore, serovars of *Salmonella* subspecies *diarizonae* may slip through undetected [19]. Hence, in addition to method ISO 6579-1 [18], it is recommended to use further indicator media when SA*S*d strains are suspected [23]. This is supported by this study as the variation also in the production of hydrogen sulphide between SA*S*d strains observed hampers the detection when xylose lysine deoxycholate agar is used.

Antimicrobial testing revealed that SA*S*d organisms from 74 sheep flocks in Thuringia were sensitive to 13 of 14 antimicrobials. All strains were resistant to only sulfamethoxazole, confirming results from earlier studies [6, 22] that did not find resistant SA*S*d strains. Therefore, the risk of transferring antimicrobial resistances via SA*S*d strains from sheep to other animal hosts or humans might be considered as negligible.

Although a wide distribution of SA*S*d was found in this study, clinical symptoms of a disease exclusively due to SA*S*d have not been detected in that period. Despite signs of rhinitis, nasal inflammation or aborted foetuses which were noted occasionally in different studies [4, 10, 11], it cannot be ruled out that factors other than SA*S*d were involved in producing these clinical signs. This occasional occurrence of clinical symptoms is also supported by the observation that experimental infections of sheep with SA*S*d indeed resulted in intestinal or nasal colonisation but not in the induction of clinical signs of a disease [24, 25]. The lack of clinical symptoms in sheep flocks harbouring SA*S*d [6, 9] has been regularly observed.

In this study, SA*S*d was detected in nearly all flocks with more than 100 sheep and even in 80 -86% of sheep flocks with only 30–100 animals. A positive correlation was found between the increasing flock size and the increasing probability of detecting SA*S*d. A higher SA*S*d prevalence in larger sheep flocks was also found in earlier studies [26] which might be due to the more successful and long-lasting persistence of SA*S*d in such flocks. Others [9] found a low within-flock prevalence regardless of the flock size, indicating that the transmission rate of the organism is limited. However, comprehensive information on the infection routes for both within the flock and from herd to herd are still missing. The role of the “ram circle”, the exchange of rams between different farms, in the transmission of SA*S*d between flocks has been discussed [9, 26], though, clear evidence is not available. Even the mode of spreading of the organism between single animals is not completely known, and detailed studies on infection routes are still needed.

To obtain information on a possible epidemiological connection and on the distribution of SA*S*d in the Thuringian sheep population macrorestriction analysis of a representative number of isolates originating from different regions and flocks was carried out. Because of the high degree of similarity in antimicrobial resistance pattern of SA*S*d and the missing correlation between biochemical index and macrorestriction pattern, only results of the macrorestriction analysis were used to generate macrorestriction groups [21] for discrimination among SA*S*d strains. The high number of genotypes revealed after digestion with both Xba*I* and Spe*I* resulted in a high number of macrorestriction groups, indicating low clonality of SA*S*d. As most macrorestriction groups were dispersed throughout the federal state and because of the lack of epidemiological data on risk factors for transmission routes it was not possible to make conclusions on an epidemiological context of SA*S*d organisms in Thuringia. Despite the more frequent occurrence of macrorestriction group C in related counties, reasons for an exchange of this SA*S*d group between the sheep herds could not be identified. Comparable data from macrorestriction studies of SA*S*d are rare [17, 22, 27] and cannot be compared directly with results of this study.

The wide distribution of SA*S*d in sheep flocks in Thuringia also raises the question on its zoonotic potential. How to deal with findings of SA*S*d in sheep flocks? In contrast to poultry, cattle and pigs, there is no regulation in place on the control of *Salmonella* infections in sheep in Germany. Despite the likely moderate to high prevalence of SA*S*d in the German sheep population, meat, meat products and cheese from sheep are very rarely contaminated with *Salmonella* organisms, SA*S*d has not been isolated from these foods [28]. Human infections caused by serovar 61: k: 1, 5, (7) have not been notified in Germany since 2000, so that the significance of SA*S*d for public health is negligible compared to that of *Salmonella enterica* subspecies *enterica* serovars [29].

Therefore, SA*S*d seemed merely to be a commensal and colonising inhabitant of the intestine and the upper respiratory tract in healthy sheep, which may occasionally become invasive only in debilitated animals. The pathogenic significance of SA*S*d as monocausal agent is considered as low since clinical signs in sheep flocks harbouring SA*S*d are, consistent to results of this study, not regularly observed [6, 9]. It is also concluded that control measures applied upon findings of SA*S*d in sheep have very little impact on reducing risks to human health and that measures to eradicate this serovar from sheep herds will probably not reduce its prevalence in the sheep population [6]. For these reasons, Sweden was the first country to make an exception for serovar SA*S*d in *Salmonella* control [6], thus reducing activities from combating to monitoring serovar SA*S*d in sheep. Despite the limited but confirming data on the disease-causing and zoonotic potential of SA*S*d, the Swedish strategy could also be a guideline for Germany, which is supported also by the results of this study. Nevertheless, given the special and interesting characteristics of SA*S*d and the persisting lack of knowledge on the infection, further studies on the pathogenicity and transmission routes of this organism in sheep will be most valuable.

## Conclusions

In the sheep population of the German federal state of Thuringia, only *Salmonella enterica* subspecies *diarizonae* serovar 61: k: 1, 5, (7) (SA*S*d) was detected in more than 80% of the examined flocks. Antimicrobial testing revealed that all SA*S*d organisms from sheep flocks in Thuringia were sensitive to 13 of 14 antimicrobials, therefore, the risk of transferring antimicrobial resistances via SA*S*d to humans can be considered as low. Despite the high prevalence of this agent, clinical symptoms of a disease exclusively due to SA*S*d have not been observed. A high number of macrorestriction groups of SA*S*d strains were found indicating a low clonality of the serovar. Data from sheep derived foods and public health data in Germany strongly suggest that the significance of SA*S*d for public health is considerably lower than that of *Salmonella enterica* subspecies *enterica* serovars. For this reason and because of the low disease-causing potential of SA*S*d in sheep, in accordance with strategies in other countries, it is worthwile to consider a reduction in the ongoing activities from combating to monitoring SA*S*d in the sheep population also in Germany.

## Abbreviations

EUCAST: European Committee on Antimicrobial Susceptibility Testing
MIC: Minimum inhibitory concentration
*S.*: *Salmonella*
SA*S*d: *Salmonella enterica* subspecies *diarizonae* serovar 61: k: 1, 5, (7)
